# Das Lymphödemrisiko von Mammakarzinompatientinnen nach Operation oder Strahlentherapie der Axilla

**DOI:** 10.1007/s00066-021-01762-9

**Published:** 2021-03-25

**Authors:** Marciana-Nona Duma

**Affiliations:** Klinik für Strahlentherapie und Radioonkologie, Universitätsklinikum Jena, Bachstr. 18, 07745 Jena, Deutschland

## Einleitung

Ziel dieser Studie war es, das Auftreten eines axillären Lymphödems bei Brustkrebspatientinnen („breast cancer-related lymphedema“ [BCRL]) in Abhängigkeit von der axillären Operationsart und der regionalen Lymphknotenbestrahlung („regional lymphnode radiation“ [RLNR]) zu vergleichen.

## Patienten und Methode

Von 2005 bis 2018 wurden 1815 Patientinnen mit invasivem Brustkrebs in eine Lymphödemscreeningstudie eingeschlossen. Die Patientinnen wurden in Abhängigkeit von der Art der axillären Behandlung in die folgenden 4 Gruppen eingeteilt: Sentinel-Lymphknoten-Biopsie (SLNB) allein, SLNB+RLNR, axilläre Lymphknotendissektion (ALND) allein und ALND+RLNR. Zur objektiven Beurteilung des Armvolumens wurde ein Perometer verwendet. Bei allen Patienten wurden präoperative Basislinienmessungen und Nachmessungen nach der Behandlung durchgeführt. Ein Lymphödem wurde definiert als eine ≥ 10 %ige relative Zunahme des Armvolumens, welche > 3 Monate postoperativ auftrat. Der primäre Endpunkt war die BCRL-Rate in den Gruppen. Sekundäre Endpunkte waren die 5‑Jahres-Lokalkontrolle und das krankheitsfreie Überleben (DFS).

## Ergebnisse

Die Kohorte umfasste 1340 Patienten mit SLNB allein, 121 mit SLNB+RLNR, 91 mit ALND allein und 263 mit ALND+RLNR. Die gesamte mediane Nachbeobachtungszeit nach der Diagnose betrug 52,7 Monate. Die kumulativen 5‑Jahres-Inzidenzraten für BCRL betrugen 30,1 %, 24,9 %, 10,7 % bzw. 8,0 % für ALND+RLNR, ALND allein, SLNB+RLNR und SLNB allein. Multivariate Cox-Modelle, die für Alter, Body-Mass-Index, Operation und Rekonstruktionstyp adjustiert waren, zeigten, dass die ALND-allein-Gruppe ein signifikant höheres BCRL-Risiko hatte (Hazard Ratio [HR] 2,66; *P* = 0,02) im Vergleich zur SLNB+RLNR-Gruppe. Es gab keinen signifikanten Unterschied im BCRL-Risiko zwischen den Gruppen ALND+RLNR und ALND allein (HR 1,20; *P* = 0,49) und zwischen den Gruppen SLNB allein und SLNB+RLNR (HR 1,33; *P* = 0,44). Die lokalen 5‑Jahres-Kontrollraten waren in den Gruppen ALND+RLNR, ALND allein, SLNB+RLNR und SLNB allein ähnlich (2,8 %, 3,8 %, 0 % bzw. 2,3 %).

## Schlussfolgerung der Autoren

Obwohl die RLNR das Risiko für ein Lymphödem des Arms auch erhöht, ist der Hauptrisikofaktor für das Auftreten eines Lymphödems die Radikalität der axillären Operation (ALND).

## Kommentar

Die Bestrahlung der Lymphabflusswege ist ein entscheidender Bestandteil der adjuvanten Therapie von Mammakarzinompatientinnen mit Lymphknotenbefall. Daten aus der MA.20- und der EORTC-22922-10925-Studie haben bewiesen, dass die zusätzliche Bestrahlung der supraklavikulären und der Mammaria-interna-Lymphabflusswege im Vergleich zur alleinigen Ganzbrust- oder Brustwandbestrahlung die regionale und Fernkontrolle sowie die krankheitsspezifische Letalität effektiv verbessert [1, 2]. Darüber hinaus zeigten die Ergebnisse der EORTC-10981-22023-AMAROS-Studie, dass die axilläre Strahlentherapie nach positiver Sentinel-node-Biopsie den Patientinnen eine ausgezeichnete axilläre Kontrolle bietet, auch wenn auf die ALND verzichtet wird [3]. Aus den MA.20- und EORTC-22922-Studien geht jedoch auch hervor, dass die BCRL-Rate-basierend auf der subjektiven klinischen Beurteilung in MA.20 mit der RLNR anstieg, und zwar von 4,5 % auf 8,4 % bzw. von 10,5 % auf 12 %. Die AMAROS-Studie zeigte, dass auch Patienten mit 1–2 befallenen axillären Lymphknoten nach ALND (ohne RLNR) oder nach SLNB, gefolgt von RLNR, ähnliche Tumorkontrollraten erreichen können (5-Jahres-Gesamtüberleben 93,3 % bzw. 92,5 %; *p* = 0,34). Darüber hinaus betrug die BCRL-Inzidenz nach 5 Jahren in der SLNB+RLNR-Gruppe 6 %, im Vergleich zu 13 % nach ALND allein. Diese Ergebnisse implizieren, dass das Risiko, ein BCRL zu entwickeln, mehr vom Ausmaß der axillären Operation als von der Bestrahlung abhängt.

Unverständlich ist uns, wieso in der Literatur nach ALND höhere Lymphödem-Raten berichtet werden (> 20 % Risiko) als in der AMAROS-Studie [4–6]. Eine mögliche Erklärung wäre, dass in AMAROS für beide Gruppen eine Kombination von ALND und RLNR zugelassen war. Wenn > 4 positive LN in einer Intention-to-treat-Analyse (ITT) vorhanden waren, kann das natürlich zu verzerrten BCRL-Raten führen, da ITT-Analysen den Effekt der Behandlungszuweisung schätzen und nicht den Behandlungseffekt. Eine andere Erklärung wäre die Methode, das Lymphödem zu quantifizieren. Welche Methode ist dafür überhaupt geeignet? Denn es ist ja bekannt, dass Umfangsänderungen der Gliedmaßen, gemessen mit einem Maßband, relativ ungenau sind und oftmals keine eindeutige Diagnose erlauben. Insbesondere können Schwellungen an verschiedenen Stellen des betroffenen Arms (z. B. an der Hand) dadurch nicht berücksichtigt werden. Andere Studien, wie z. B. IBCSG23-01, haben nach CTCAE nur eine Beurteilung des BCRL durch den Arzt erlaubt. Das bedeutete vermutlich mehr Subjektivität bei der BCRL-Diagnose und könnte die unterschiedlichen Angaben in der Literatur erklären [7]. Bekannt ist auch, dass ein Perometer bei der Lymphödemeinschätzung zuverlässiger als eine Armumfangsmessung ist und auch deshalb oft als Screeningmethode empfohlen wird [8, 9].

Die in der hier kommentierten Studie beschriebenen prospektiven Methoden versuchen, die Fallstricke bei der Beurteilung von Lymphödemen zu umgehen. Darüber hinaus wird durch die Verwendung der RVC- oder WAC-Formel eine Kontrolle eingeführt, die Änderungen des Volumens des betroffenen Arms im Verhältnis zum kontralateralen Arm (RVC) oder zum Körpergewicht (WAC) beurteilt. Von ihnen ist bekannt, dass sie während und nach der Behandlung schwanken. Dadurch haben die Autoren der hier kommentierten Studie die Objektivität bei der Lymphödemeinschätzung erhöht und eine aussagekräftige Antwort auf die Frage der „schuldigen“ Behandlungsmethode gegeben.

## Fazit

Die hier diskutierte Studie konnte die unterschiedlichen BCRL-Raten in den verschiedenen Behandlungsgruppen sehr gut darstellen („breast cancer-related lymphedema“ [BCRL]) mit SLNB allein (8 %), SLNB+RLNR (10,7 %), ALND (24,9 %) und ALND+RLNR (30,1 %). Obwohl die Patienten, die SLNB/ALND+RLNR erhielten, ein gering höheres Lymphödemrisiko hatten im Vergleich zu denen mit SLNB/ALND allein, war dieser Unterschied bei der multivariablen Analyse nicht signifikant. Der Hauptrisikofaktor für das BCRL bleibt also das Ausmaß der axillären Operation und nicht die zusätzliche RLNR. Das bestätigt die früheren AMAROS- und IBCSG23-01-Ergebnisse und unterstreicht den Nutzen der adjuvanten (regionalen Lymphknotenbestrahlung [RLNR]), die ohne eine zusätzliche signifikante Toxizität durchgeführt werden kann.

*Marciana-Nona Duma, Jena*

## References

[1] Poortmans PM (2015). Internal mammary and medial supraclavicular irradiation in breast cancer. N Engl J Med.

[2] Whelan TJ (2015). Regional nodal irradiation in early-stage breast cancer. N Engl J Med.

[3] Donker M (2014). Radiotherapy or surgery of the axilla after a positive sentinel node in breast cancer (EORTC 10981-22023 AMAROS): a randomised, multicentre, open-label, phase 3 non-inferiority trial. Lancet Oncol.

[4] Gillespie TC (2018). Breast cancer-related lymphedema: risk factors, precautionary measures, and treatments. Gland Surg.

[5] Giuliano AE (2017). Effect of axillary dissection vs no axillary dissection on 10-year overall survival among women with invasive breast cancer and sentinel node metastasis the ACOSOG Z0011 (alliance) randomized clinical trial. JAMA.

[6] Liljegren G, Holmberg L (1997). Arm morbidity after sector resection and axillary dissection with or without postoperative radiotherapy in breast cancer stage. 1. Results from a randomised trial. Eur J Cancer.

[7] Galimberti V (2013). Axillary dissection versus no axillary dissection in patients with sentinel-node micrometastases (IBCSG 23-01): a phase 3 randomised controlled trial. Lancet Oncol.

[8] Dylke ES, Ward LC, Kilbreath SL (2013). Standardized approach to lymphedema screening. Oncologist.

[9] Ancukiewicz M (2011). Standardized method for quantification of developing lymphedema in patients treated for breast cancer. Int J Radiat Oncol Biol Phys.

